# AI in medical education: medical student perception, curriculum recommendations and design suggestions

**DOI:** 10.1186/s12909-023-04700-8

**Published:** 2023-11-09

**Authors:** Qianying Li, Yunhao Qin

**Affiliations:** 1 Department of Orthopedics, Shanghai Sixth People’s Hospital, Shanghai Jiao Tong University, Shanghai, China; 2 Antai College of economics and management, Shanghai Jiao Tong University, Shanghai, China

**Keywords:** Medical AI, Artificial Intelligence, UTAUT2, Medical training, Medical students

## Abstract

Medical AI has transformed modern medicine and created a new environment for future doctors. However, medical education has failed to keep pace with these advances, and it is essential to provide systematic education on medical AI to current medical undergraduate and postgraduate students. To address this issue, our study utilized the Unified Theory of Acceptance and Use of Technology model to identify key factors that influence the acceptance and intention to use medical AI. We collected data from 1,243 undergraduate and postgraduate students from 13 universities and 33 hospitals, and 54.3% reported prior experience using medical AI. Our findings indicated that medical postgraduate students have a higher level of awareness in using medical AI than undergraduate students. The intention to use medical AI is positively associated with factors such as performance expectancy, habit, hedonic motivation, and trust. Therefore, future medical education should prioritize promoting students’ performance in training, and courses should be designed to be both easy to learn and engaging, ensuring that students are equipped with the necessary skills to succeed in their future medical careers.

**Supplementary Information:**

The online version contains supplementary material available at 10.1186/s12909-023-04700-8.

## Introduction

In 1956, John McCarthy introduced the concept of artificial intelligence (AI) during the Dartmouth conference, marking the inception of this field [1]. Kaplan and Haenlein later defined AI as “the ability to process external data systematically and learn from it to achieve specific goals and tasks” [2]. The idea of using AI in medicine emerged in the early 1970s to enhance medical diagnosis and treatment. In recent years, there have been remarkable advancements in AI, leading to the development of medical AI systems capable of diagnosing diseases with expert-level accuracy [3]. This has brought about a revolution in medicine, improving healthcare services and promoting human health. The future of medical AI is expected to be even more promising, with potential applications including personalized treatment plans, drug development, and virtual healthcare assistants [4]. In China, some medical AIs have also been applied to daily clinic practice, such as in management of medical record and biobank information [5], disease screening [6–8] and diagnosing [9–11]. Despite increasing interest in this technology, medical education has not kept pace with the remarkable breakthroughs made in AI [12]. Although there have been calls to action, the adoption of AI training into medical education has been limited. As the adoption of AI continues to grow in healthcare, integration into medical education could offer substantial benefits for future practice, as medical education can reach the largest group of medical trainees early in their careers. Previous studies have shown that medical students are often not familiar with AI and may be worried about the potential for job loss [13–15]. However, they are generally enthusiastic about learning and using AI in their practice [16, 17]. Moreover, there is currently a lack of research exploring the significance of medical AI within the context of medical education in China. Therefore, understanding the attitudes of medical students toward medical AI is urgently needed to ensure the effective integration of this technology into medical education.

However, there has been limited research on the acceptance and intention to use medical AI among medical undergraduate and postgraduate students. To address this gap, we select and implement a unified theory of acceptance and use of technology 2 (UTAUT2) model in this study. The UTAUT2 model is a commonly used theoretical framework for identifying potential factors that influence acceptance [18]. Its core principle is that the intention to use technology directly predicts actual usage [19]. While the UTAUT2 model is originally developed for the workplace context, it has also been successfully applied to other domains such as internet banking, digital education, and online gaming, as well as in the medical sector, such as for the adoption of electronic medical records [20–23], clinical decision support systems [24], and disease monitoring and management applications [25]. As the UTAUT2 model has demonstrated generalizability, the determinants of acceptance identified by the model may also serve as promising predictors for acceptance of medical AI.

Given the significant and ongoing impact of medical AI on the practice of modern medicine, it is essential to integrate its use into medical education to enhance students’ performance in training and career development. This study investigated student perception of medical AI by UTAUT2 model, and made suggestion for future medical education and curriculum design.

## Materials and methods

### Survey design

This study was a survey based on UTAUT2 model conducted in 13 universities and 33 hospitals. The survey comprised three sections. The first section informed all participants about the study’s purpose, their right to withdraw at any time, and that their data would be collected anonymously. The survey concluded when participants declined to participate. The second section collected demographic data, including participants’ school of medicine, age, sex, year of medical education, experience with AI, and self-evaluation of their experience of medical AI. The survey concluded when participants self-assessed themselves without experience of using AI or medical AI. The third section contained 38 5-point Likert questions focused on 10 factors: effort expectancy, performance expectancy, social influence, hedonic motivations, price value, habit, facilitating condition, behavioral intention, technology fear, and technology trust. These questions were designed to assess medical students’ acceptance and intention to use medical AI. The survey was conducted online using Wen Juan Xing (www.wjx.cn) via WeChat and the web. The Likert scale used to quantify different dimensions or constructs ranged from 0 (strongly disagree) to 4 (strongly agree). The link of survey was sent by teachers to students during class.

### Settings

The Chinese medical students started to learn medicine upon entering university. Following a comprehensive 5-year medical education, these undergraduate students had the opportunity to advance their medical knowledge by gaining admission to a postgraduate school of medicine. This allows them to pursue further specialized medical education and training. Or they can take a 3-year training to become general practitioners.

### Hypothesis

Based on the UTAUT2 model, we formulated 10 hypotheses to test key factors that exerted a significant influence on the acceptance and intention to use of medical AI among both medical undergraduate and postgraduate students.

The hypotheses were listed below:

#### H1

Performance expectancy has a positive impact on behavioral intention towards the use of medical AI.

#### H2

Effort Expectancy has a positive impact on behavioral intention towards the use of medical AI.

#### H3

Social Influence has a positive impact on behavioral intention towards the use of medical AI.

#### H4

Hedonic Motivation have a positive impact on behavioral intention towards the use of medical AI.

#### H5

Price Value has a positive impact on behavioral intention towards the use of medical AI.

#### H6

Habit has a positive impact on behavioral intention towards the use of medical AI.

#### H7

Facilitating Conditions has a positive impact on the behavioral intention towards use of medical AI.

#### H8

Technology Fear has a negative impact on the behavioral intention towards use of medical AI.

#### H9

Trust has a positive impact on the behavioral intention towards use of medical AI.

#### H10

Behavioral intention has a positive effect on usage behavior in distance education systems.

### Participants

(1) Medical undergraduate students in their fourth and fifth years of study were included in this research. (Fourth-year undergraduate students were mandated to complete a year-long hospital internship). (2) All grade of medical postgraduate students were included. (3) No absent information in participants’ school of medicine, age, sex, year of medical education, experience with AI, and self-evaluation of their experience of medical AI.

### Statistical method

Partial least squares were used to analyze the path coefficients, which indicate the relationships between the dependent and independent variables. To measure the data’s internal reliability, Cronbach’s α was evaluated against the standard threshold of 0.7, the criterion for acceptable internal consistency of data. Convergent validity was calculated using average variance extracted (AVE) and composite reliability (CR). The discriminant validity of the measurement model was analyzed by using the restrictive method of the Heterotrait-Monotrait (HTMT) ratio to ensure that all values were below 0.9. The data analysis was conducted by using SmartPLS 4.0. The basic information of age, sex, year of medical education, experience with AI was analysis by chi-square test (SPSS 26.0).

## Results

During a 2-week period, 1,243 undergraduate and postgraduate medical students from 13 universities and 33 hospitals completed the questionnaire. Of these, 41.5% (516) were male and 58.5% were female, 66.8% (830) were undergraduates and 33.2% (413) were postgraduates. The mean age was 24.9 ± 5.7 years old. Respondents were asked whether they were aware of using AI equipment or systems in their daily lives, and 199 respondents said they did not use AI equipment or software. Among the remaining 1,044 respondents, 369 had no experience of using medical AI. There was no significant difference between sex and self-evaluation of their experience of using AI and medical AI (χ2 = 0.02, p = 0.886). However, a significantly higher proportion of postgraduates (75.0%) reported had experience of using medical AI compared to undergraduates (59.5%, χ2 = 24.4, p < 0.001). Finally, a total of 675 respondents with experience of medical AI completed the survey.

**Fig. 1:**
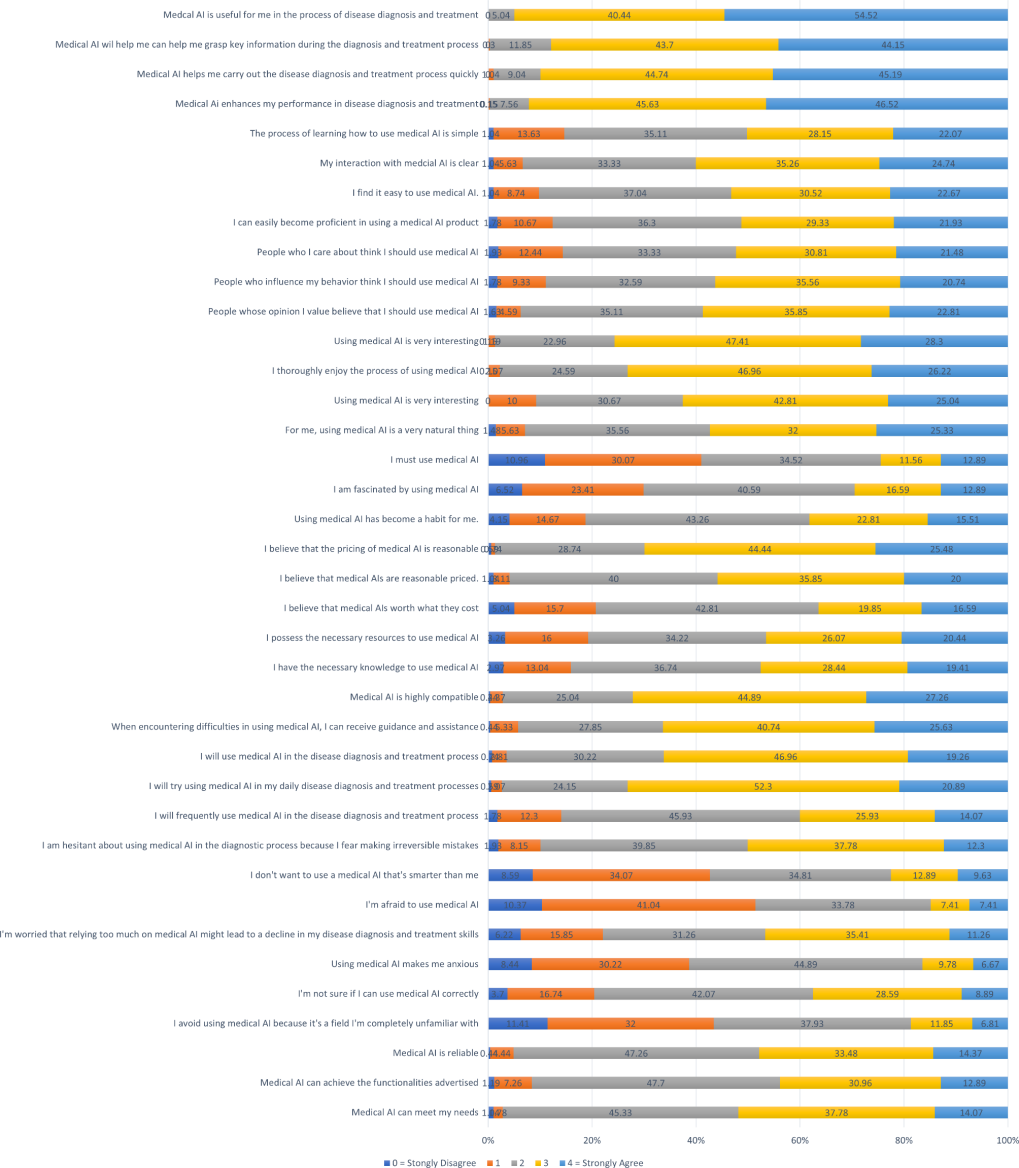
The responses for each measurement scale

Figure 1 displayed the responses for each measurement scale. Table 1 showed the calculated values of Cronbach’s α, which ranged from 0.833 to 0.916, while CR varied in the range of 0.895–0.947 and AVE ranged from 0.623 to 0.856. To assess the discriminant validity of the measurement model, we used the restrictive method of the HTMT, which ensured that all values were below 0.9. (Table 2)

**Table 1:** Construct reliability and convergent validity

	Item		Cronbach’s alpha	(rho_a)	Composite Reliability	AVE
BI	3		0.875	0.888	0.923	0.800
EE	4		0.908	0.911	0.936	0.785
FC	4		0.843	0.843	0.895	0.680
HM	3		0.916	0.919	0.947	0.856
HT	4		0.868	0.867	0.911	0.720
PE	4		0.911	0.912	0.938	0.790
PV	3		0.833	0.843	0.900	0.751
SI	3		0.882	0.897	0.927	0.808
TF	7		0.904	0.945	0.920	0.623
TR	3		0.878	0.883	0.925	0.804

BI: behavioral intention; EE: effort expectancy; FC: facilitating condition; HM: hedonic motivations; HT: habit; PE: performance expectancy; PV: price value; SI: social influence; TF: technology fear; TR: technology trust

**Table 2:** Discriminant Validity (Ratio Heterotrait-Monotrait -HTMT)

	BI	EE	FC	HM	HT	PE	PV	SI	TF	TR
BI										
EE	0.572									
FC	0.710	0.799								
HM	0.722	0.661	0.787							
HT	0.830	0.665	0.777	0.658						
PE	0.568	0.523	0.569	0.598	0.452					
PV	0.668	0.657	0.846	0.784	0.741	0.548				
SI	0.615	0.642	0.827	0.634	0.663	0.500	0.715			
TF	0.217	0.210	0.289	0.128	0.346	0.056	0.245	0.343		
TR	0.808	0.635	0.796	0.713	0.848	0.524	0.818	0.684	0.284	

BI: behavioral intention; EE: effort expectancy; FC: facilitating condition; HM: hedonic motivations; HT: habit; PE: performance expectancy; PV: price value; SI: social influence; TF: technology fear; TR: technology trust

**Fig. 2:**
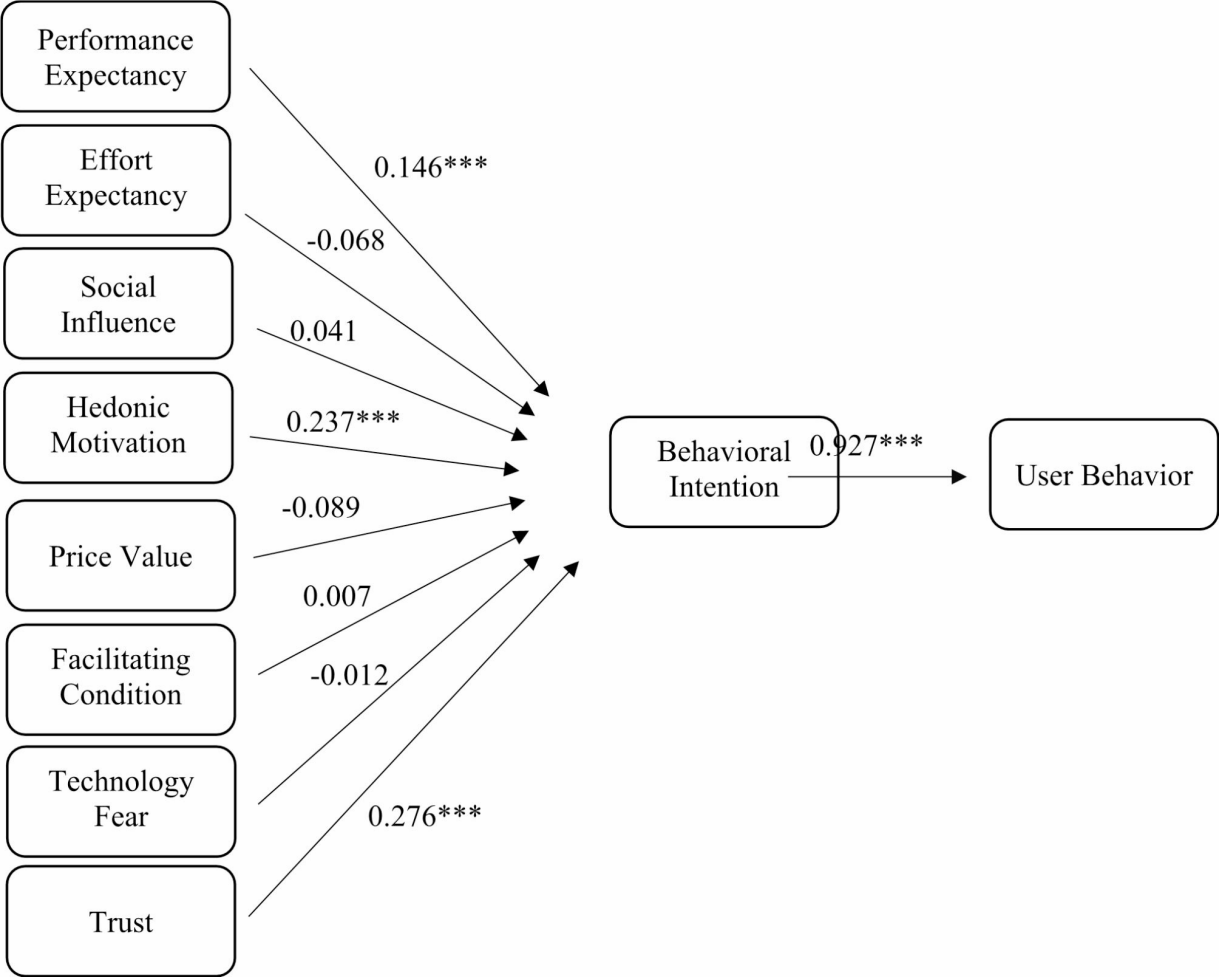
Path ecoefficiency analysis. ***, P < 0.001

The results of the hypothesis testing for the standardized path coefficients and path significance are presented in Table 3; Fig. 2, which showed that performance expectancy, hedonic motivation, habit, and trust had a positive influence on the intention to use medical AI.

**Table 3:** Structural Model Analysis (Path coefficients)

	Original Samples	p-values
H1. Performance Expectancy → Behavioral Intention	0.146	0.000***
H2. Effort Expectancy → Behavioral Intention	-0.068 (ns)	0.101
H3. Social Influence → Behavioral Intention	0.041 (ns)	0.346
H4. Hedonic Motivation → Behavioral Intention	0.237	0.000***
H5. Price Value → Behavioral Intention	-0.089 (ns)	0.068
H6. Habit → Behavioral Intention	0.388	0.000***
H7. Facilitating Conditions → Behavioral Intention	0.007 (ns)	0.895
H8. Technology Fear → Behavioral Intention	-0.012 (ns)	0.585
H9. Technology Trust → Behavioral Intention	0.276	0.000***
H10. Behavioral Intention → User Behavior	0.927	0.000***

ns: Not significant. ***p < 0.001

## Discussion

This study aims to investigate the factors influencing the acceptance of medical artificial intelligence (AI) among undergraduate and postgraduate medical students, using the UTAUT2 framework. The study identifies performance expectancy, hedonic motivation, habit, and trust as the primary drivers that impact the acceptance and utilization of medical AI.

Performance expectancy refers to individual’s belief that utilizing a specific system would be more beneficial to him/her and would improve the performance of the task. Our results were consistent with previous research, particularly in the context of e-learning environments. For instance, Oye et al. found that the performance expectancy of medical educators positively impacted their acceptance and use of information technologies in the workplace [26]. Hedonic motivation refers to the user’s pleasure derived from utilizing a system. The confirmation of hedonic motivation in this study strongly indicates its positive influence on behavioral intention. These findings align with the results reported by Tarhini, et al. and Moorthy, et al. [27, 28]. Habit refers to the automatic or habitual behavior individuals develop in their use of technology. The significance of habit as a predictor of behavioral intention has been highlighted in previous research as well [29, 30]. Venkatesh et al. demonstrated that the routine use of a technology had a notable impact on its adoption [18]. Trust represents another crucial factor driving the acceptance and intention to use medical AI. Previous studies have consistently shown significant direct relationships between trust and behavioral intention, trust and attitude, and trust and usage intention. Cabrera-Sánchez et al. were pioneers in introducing trust into the UTAUT2 model, and their research demonstrated the significant role of trust in shaping consumer behavioral intent to use AI applications [19].

### For the future medical AI education and design

Our findings revealed that only 675 (54.3%) participants self-reported familiarity with medical AI, indicating that nearly half of the medical students were not acquainted with this field. Furthermore, a limited number of medical schools in China had incorporated medical AI into their curriculum for students. In addition, we found performance expectancy, hedonic motivation, habit, and trust were key factors that affected the use of medical AI. Given these outcomes, we propose the following recommendations for **the future medical AI education**::

1. **Enhance Awareness**: Given the considerable proportion of students unfamiliar with medical AI, efforts should be directed towards heightening awareness through educational activities.
2. **Curriculum Enrichment**: Collaborative efforts between academia and industry should be pursued to integrate medical AI topics into the curriculum, ensuring students are well-versed in this evolving field.
3. **Addressing Student Needs**: Focus on training that addresses students’ performance expectancy, hedonic motivation, habit formation, and trust, as these emerged as crucial factors influencing medical AI adoption. (1) For performance expectancy, future medical AI education should prioritize teaching students how to leverage its use to improve their training outcomes and future careers. (2) For habit and hedonic motivation, teacher should deliver enjoyable and entertaining experience when utilizing medical AI. (3) For trust, it is important for teachers to instruct students on how to assess the reliability and credibility of medical AI systems.

### For the future design of medical AI

1. **Enhancing Performance**: medical AI should emphasize assisting students in gathering critical medical information, streamlining tasks with speed and precision, and enhancing overall performance.
2. **User Friendly and Engaging**: Considering the importance of hedonic motivation and habit, students anticipate an enjoyable and entertaining experience when utilizing medical AI. Interactions involving issuing commands, asking questions, and receiving prompt replies or necessary information contribute to a sense of hedonic pleasure, enhancing the engagement and appeal of the technology.
3. **Establishing Trust**: Trust plays a crucial role in the acceptance and utilization of medical AI among medical students. Medical AI systems that offer the latest disease guidelines or cutting-edge medical knowledge are likely to be well-received by students.

### Limitations

Despite its contributions, our study has several limitations that should be considered. Firstly, the online survey method and voluntary participation may have introduced a bias by only attracting students interested in the subject matter. Secondly, while our data was collected from 33 hospitals and 11 universities in seven provinces of China, caution should be exercised when generalizing the results to the entire country. Thirdly, even though AI has been applied in clinical medicine for some time, medical undergraduates and postgraduates in China have not yet received systematic education on medical AI. Consequently, the perspectives conveyed by the participants are inevitably shaped by their interactions with the specific medical AI tools they engage with in their routine practices. This variation in exposure has inherently led to a spectrum of attitudes towards different medical AI applications. Future studies could further investigate the intricate relationship between a specific and universally embraced medical AI education approach and its potential to enhance students’ academic performance.

## Conclusion

With the increasing prevalence of AI technologies in the field of medicine, it is evident that future medical undergraduate and postgraduate students will operate within a different professional landscape. However, despite this paradigm shift, students currently lack structured and standardized education on medical AI, which can leave them feeling uninformed and unprepared. This study has identified key factors that influence the acceptance and utilization of medical AI among students, emphasizing the imperative for future education that focuses on promoting performance in training and career through AI adoption, as well as the ability to discern the reliability and credibility of AI systems. It is crucial to design medical AI courses that are both user-friendly and engaging, ensuring that students acquire the essential skills to thrive in their forthcoming medical careers.

### Electronic supplementary material

Below is the link to the electronic supplementary material.

Supplementary Material 1

## Data Availability

The datasets and analyzed during the present research are available from the corresponding author on reasonable request.
