# Supplementary material for: AI in medical education: medical student perception, curriculum recommendations and design suggestions
Source: BMC Med Educ. 2023 Nov 9;23:852. doi: 10.1186/s12909-023-04700-8 (PMC10637014; doi:10.1186/s12909-023-04700-8)
Supplement: Supplementary file 1 — Supplementary Material 1 [file 12909_2023_4700_MOESM1_ESM.docx]

Hello! We sincerely appreciate the time you've dedicated to completing this questionnaire. The purpose of this questionnaire is to explore the level of understanding and attitude of medical students towards artificial intelligence (AI) technology in the field of medicine. Please answer based on your genuine thoughts and understanding of the questions. We will strictly keep your information and data confidential. Thank you for your participation!

AI technology has been widely used in the field of medicine, giving rise to many mature medical AI software and hardware systems. Notable examples encompass IBM Watson Health, Google Health/DeepMind-assisted diagnostic systems, IRIS.AI literature assistance system, XtalPi's aided drug design system, the da Vinci surgical robot, among others.

Against this backdrop, we would like to ask you to answer the following questions:

Q1. Sex:

1. Male.
2. Female.

Q2. Your Age

Q3. What level of education are you currently pursuing?

1. Undergraduate.
2. Postgraduate.

Q4. The university or hospital you are affiliated with.

Q5. Have you come into contact with AI products in daily life (such as smart devices, software, etc.)?

1. Yes.
2. No.

Q6. Have you heard of or used any of the following "any one or similar" medical AI products: da Vinci/Tianji surgical robots, integrated smart diagnosis and triage machines, smart wearable devices, Aidoc intelligent imaging, iCAD intelligent breast cancer diagnosis system, Vizier.AI intelligent stroke diagnosis system, IBM Watson Health, Google Health/DeepMind assisted diagnostic systems, IRIS.AI literature assistance system, XtalPi assisted drug design system, etc.?

1. Yes.
2. No.

From intelligent surgical robots to medical wearable devices and autonomous mobile robots (such as intelligent diagnosis and triage machines), medical AI has entered our learning, work, and daily life. Medical AI covers a wide range of areas, from protein structure prediction and drug development for basic biological research to clinical-assisted diagnostic and treatment systems.

Medical AI comprehensively encompasses "disease prevention and screening," "disease diagnosis," and "disease treatment." In terms of disease screening, medical AI rapidly identifies positive cases, saving time for medical professionals. In disease diagnosis, medical AI can provide cutting-edge information on disease treatment, assisting healthcare personnel in decision-making. In disease treatment, AI-assisted surgical robots in the medical field can reduce surgical damage and shorten operation times.

In this context, what is your opinion?

Q1. I believe that medical AI is useful in the process of disease diagnosis and treatment.

0) Total Disagree. 1) Disagree. 2) Normal. 3) Agree. 4) Total Agree.

Q2. I believe that medical AI can help me grasp key information during the diagnosis and treatment process.

0) Total Disagree. 1) Disagree. 2) Normal. 3) Agree. 4) Total Agree.

Q3. I believe that medical AI helps me carry out the disease diagnosis and treatment process quickly.

0) Total Disagree. 1) Disagree. 2) Normal. 3) Agree. 4) Total Agree.

Q4. I believe that medical AI enhances my performance in disease diagnosis and treatment.

0) Total Disagree. 1) Disagree. 2) Normal. 3) Agree. 4) Total Agree.

Q5. The process of learning how to use medical AI is simple.

0) Total Disagree. 1) Disagree. 2) Normal. 3) Agree. 4) Total Agree.

Q6. Medical AI is clear and easy to understand.

0) Total Disagree. 1) Disagree. 2) Normal. 3) Agree. 4) Total Agree.

Q7. I find it easy to use medical AI.

0) Total Disagree. 1) Disagree. 2) Normal. 3) Agree. 4) Total Agree.

Q8. I can easily become proficient in using a medical AI product.

0) Total Disagree. 1) Disagree. 2) Normal. 3) Agree. 4) Total Agree.

There are various types of medical AI (as mentioned earlier, including surgical robots, medical wearable smart devices, diagnostic and treatment assistance systems, bioinformatics analysis and prediction, etc.). In what situations do you think you would use medical AI?

Q9. People who I care about think I should use medical AI.

0) Total Disagree. 1) Disagree. 2) Normal. 3) Agree. 4) Total Agree.

Q10. People who influence my behavior think I should use medical AI.

0) Total Disagree. 1) Disagree. 2) Normal. 3) Agree. 4) Total Agree.

Q11. People whose opinion I value believe that I should use medical AI.

0) Total Disagree. 1) Disagree. 2) Normal. 3) Agree. 4) Total Agree.

Q12. I possess the necessary resources to use medical AI.

0) Total Disagree. 1) Disagree. 2) Normal. 3) Agree. 4) Total Agree.

Q13. I have the necessary knowledge to use medical AI.

0) Total Disagree. 1) Disagree. 2) Normal. 3) Agree. 4) Total Agree.

Many people have already begun using medical AI, from smartwatches that can monitor blood pressure and heart rate to surgical robots used in operating rooms. Medical AI is quietly changing the way we learn, work, and live.

In this context, may I ask?

Q14. Medical AI is highly compatible.

0) Total Disagree. 1) Disagree. 2) Normal. 3) Agree. 4) Total Agree.

Q15. When encountering difficulties in using medical AI, I can receive guidance and assistance.

0) Total Disagree. 1) Disagree. 2) Normal. 3) Agree. 4) Total Agree.

Q16. I believe that the pricing of medical AI is reasonable.

0) Total Disagree. 1) Disagree. 2) Normal. 3) Agree. 4) Total Agree.

Q17. Using medical AI is very interesting.

0) Total Disagree. 1) Disagree. 2) Normal. 3) Agree. 4) Total Agree.

Q18. Using medical AI is a pleasurable experience.

0) Total Disagree. 1) Disagree. 2) Normal. 3) Agree. 4) Total Agree.

Q19. I thoroughly enjoy the process of using medical AI.

0) Total Disagree. 1) Disagree. 2) Normal. 3) Agree. 4) Total Agree.

Q20. I believe that medical AIs are reasonable priced.

0) Total Disagree. 1) Disagree. 2) Normal. 3) Agree. 4) Total Agree.

Q21. I believe that medical AIs worth what they cost.

0) Total Disagree. 1) Disagree. 2) Normal. 3) Agree. 4) Total Agree.

Q22. Using medical AI has become a habit for me.

0) Total Disagree. 1) Disagree. 2) Normal. 3) Agree. 4) Total Agree.

Q23. I am fascinated by using medical AI.

0) Total Disagree. 1) Disagree. 2) Normal. 3) Agree. 4) Total Agree.

Q24. I must use medical AI.

0) Total Disagree. 1) Disagree. 2) Normal. 3) Agree. 4) Total Agree.

Q25. Medical AI is reliable.

0) Total Disagree. 1) Disagree. 2) Normal. 3) Agree. 4) Total Agree.

Q26. Medical AI can achieve the functionalities advertised.

0) Total Disagree. 1) Disagree. 2) Normal. 3) Agree. 4) Total Agree.

Q27. Medical AI can meet my needs.

0) Total Disagree. 1) Disagree. 2) Normal. 3) Agree. 4) Total Agree.

With the iterative upgrades of AI technology, medical AI will become more diverse and feature rich. In the face of this trend, will you consider in the future...

Q28. I will use medical AI in the disease diagnosis and treatment process.

0) Total Disagree. 1) Disagree. 2) Normal. 3) Agree. 4) Total Agree.

Q29.I will try using medical AI in my daily disease diagnosis and treatment processes.

0) Total Disagree. 1) Disagree. 2) Normal. 3) Agree. 4) Total Agree.

Q30. I will frequently use medical AI in the disease diagnosis and treatment process.

0) Total Disagree. 1) Disagree. 2) Normal. 3) Agree. 4) Total Agree.

Q31. For me, using medical AI is a very natural thing.

0) Total Disagree. 1) Disagree. 2) Normal. 3) Agree. 4) Total Agree.

Q32. I am hesitant about using medical AI in the diagnostic process because I fear making irreversible mistakes.

0) Total Disagree. 1) Disagree. 2) Normal. 3) Agree. 4) Total Agree.

Q33. I don't want to use a medical AI that's smarter than me.

0) Total Disagree. 1) Disagree. 2) Normal. 3) Agree. 4) Total Agree.

Q34. I'm afraid to use medical AI.

0) Total Disagree. 1) Disagree. 2) Normal. 3) Agree. 4) Total Agree.

Q35. I'm worried that relying too much on medical AI might lead to a decline in my disease diagnosis and treatment skills.

0) Total Disagree. 1) Disagree. 2) Normal. 3) Agree. 4) Total Agree.

Q36. Using medical AI makes me anxious.

0) Total Disagree. 1) Disagree. 2) Normal. 3) Agree. 4) Total Agree.

Q37. I'm not sure if I can use medical AI correctly.

0) Total Disagree. 1) Disagree. 2) Normal. 3) Agree. 4) Total Agree.

Q38. I avoid using medical AI because it's a field I'm completely unfamiliar with

0) Total Disagree. 1) Disagree. 2) Normal. 3) Agree. 4) Total Agree.
